# Multiple Myeloma in a Young Female Presenting with Neurological Symptoms

**DOI:** 10.1155/2020/1375174

**Published:** 2020-02-13

**Authors:** Matthew Lee, Xinmin Zhang, Vinh Nguyen, Monique Hartley-Brown

**Affiliations:** ^1^Internal Medicine Resident, Donald and Barbara Zucker School of Medicine at Hofstra/Northwell, 300 Community Dr, Manhasset, NY 11030, USA; ^2^Donald and Barbara Zucker School of Medicine at Hofstra/Northwell, Pathology Department at North Shore University Hospital-Long Island Jewish Medical Center, 300 Community Dr, Manhasset, NY 11030, USA; ^3^Donald and Barbara Zucker School of Medicine at Hofstra/Northwell, Radiology Department at North Shore University Hospital-Long Island Jewish Medical Center, 300 Community Dr, Manhasset, NY 11030, USA; ^4^Donald and Barbara Zucker School of Medicine at Hofstra/Northwell, Hematology-Oncology Department at North Shore University Hospital-Long Island Jewish Medical Center, 300 Community Dr, Manhasset, NY 11030, USA

## Abstract

**Background:**

Multiple myeloma is overall the 14^th^ most common malignancy but is rarely seen in those younger than 35 years. Those in the younger age group have been shown to have a more aggressive course but reportedly have had similar responses to treatment compared to older cohorts. Extramedullary plasmacytomas are discrete soft tissue masses of neoplastic monoclonal plasma cells that often occur in patients with multiple myeloma.

**Case:**

This case entails a young female with new diagnosis of multiple myeloma and multiple extramedullary plasmacytomas presenting with neurological symptoms. She was treated with CyBorD regimen but was refractory and progressed. Treatment was changed to DCEP regimen, and daratumumab was added for primary refractory myeloma. The patient improved rapidly.

**Conclusion:**

This is a unique case of multiple myeloma in a young female that had failed first-line treatment but responded to targeted therapy of daratumumab. It highlights that multiple myeloma may present atypically in young patients. More research is needed on appropriate initial diagnosis and treatment of patients in this age group.

## 1. Introduction

Multiple myeloma is a malignant B-cell disorder that leads to proliferative plasma cells. It is the 14^th^ most common malignancy accounting for 1.8% of all malignancies and 10% of hematologic malignancies [1]. However, multiple myeloma rarely occurs in those younger than 35 years (0.5%) [1]. Prior studies have shown a more aggressive presentation in this age group with higher rates of extramedullary plasmacytomas, plasma cell leukemias (11%), osteolytic lesions, renal failure (25%), and Bence Jones proteinuria (81%) [2–10]. This could be explained by either a delay in diagnosis or the inherent aggressive nature of disease in younger patients [11]. It has also been reported that this younger age group has had similar overall survival and remission to older cohorts [2–5, 11].

Here, we present a case of a young female newly diagnosed with multiple myeloma. She initially presented with significant neurological symptoms and multiple extramedullary plasmacytomas throughout the body including intracranially. She received first-line treatment with combined conventional chemotherapy and proteasome inhibitor (bortezomib), but was refractory. Second-line intensive chemotherapy initially failed, but with the addition of single agent daratumumab, the patient had a favorable disease response.

## 2. Case

A 24-year-old Egyptian female presents after a syncopal fall and head trauma. She reports having multiple falls for the past month prior to admission. Upon evaluation, the patient reported neurological symptoms including left-sided weakness in the upper and lower extremities, diplopia, and ataxia along with dysphagia. Most pertinent neurological exam findings were left abducens (CN VI) palsy, bilateral upper and lower extremity weakness of 2/5, and inability to stand due to pain with a 1 cm palpable left soft tissue pelvic mass.

**Figure 1:**
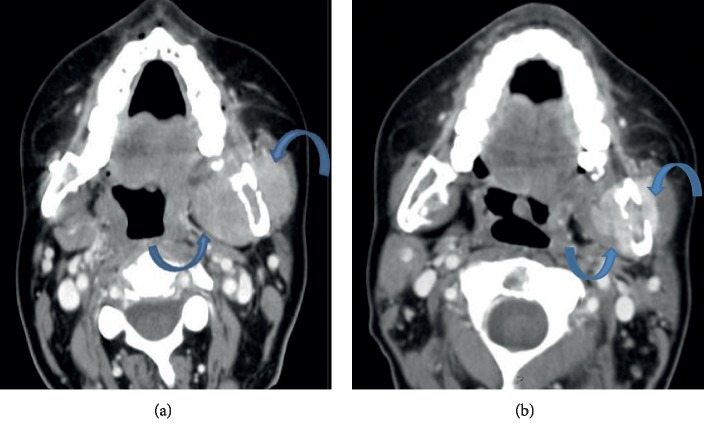
Soft tissue masses of the left masticator space seen on admission is associated with adjacent mandibular erosion (a). This progressed and increased in size during admission, but finally showed response on CDEP and daratumumab (b).

**Figure 2:**
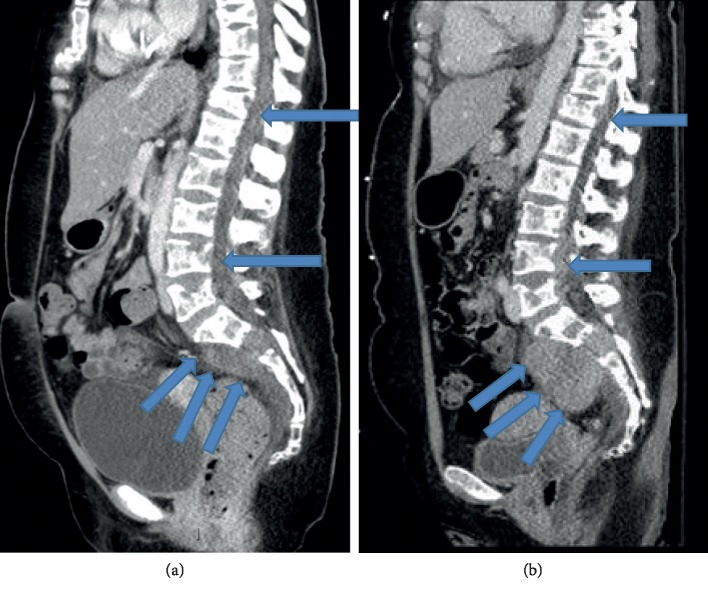
Admission imaging (a) revealed multiple paraspinal and epidural masses (arrows) which decreased (b) following DCEP and daratumumab.

Initial lab work revealed an elevated lactate dehydrogenase 375 U/L. The CBC and CMP findings were otherwise normal. Previous imaging from Egypt showed a fracture of her mandible with bilateral plate placement on X-ray. On our imaging, CT brain and maxillofacial showed several soft tissue masses present with contiguous bony erosion and destruction involving the central skull base and mandible (Figure 1(a)). At the skull base, the soft tissue mass eroded the basisphenoid and bordering maxilla, as well as the left greater wing of the sphenoid into the middle cranial fossa with infiltration of the left lateral rectus in the left orbit. CT of the spine showed paraspinal and epidural masses with bordering vertebral erosion (Figure 2(a)). CT of the chest, abdomen, and pelvis with contrast showed multiple lesions, with soft tissue infiltrating the chest wall, and left hemipelvis straddling the sacrum to involve the left gluteus musculature. These were superimposed on diffusely lytic lesions throughout thoracolumbar spine, some associated with mild compression fractures. MRI of the brain, cervical, thoracic, and sacral spine showed decreased T1 and T2 along with hyperintensive signals on diffusion of the calvarium, skull base, and facial bones with lytic lesions. Large expansile lesion involves L lateral orbital bony margin and zygoma 11 mm × 5 mm × 1.2 cm concerning for neoplasm. No new ischemic changes were observed in the brain. Cervical spine shows irregular T1 enhancement in clivus, C1, and C2, diffusely throughout C3-T1, and there was no central canal or foraminal compromise. Thoracic spine with extensive metastatic lesions with multiple compressions most pronounced at T4, T6, T8, and T11 and leptomeningeal involvement. Lumbar spine showing compression upon the cauda equina at the levels of L3-L4 and L4-L5 soft tissue in the left L3-L4, L4-L5, and L5-S1 neural foramina, encasing/compressing the left L3, L4, and L5 nerve root and bulky bilateral sacral masses.

At the time of initial evaluation, the main differential diagnosis was metastatic solid tumor malignancy, atypical neuroblastomas, and lymphoma. Decadron 4 mg every 6 hours was given for empiric treatment to reduce paraspinal inflammation. A fine needle aspiration/biopsy was done via CT guidance of a soft tissue sternal mass.

**Figure 3:**
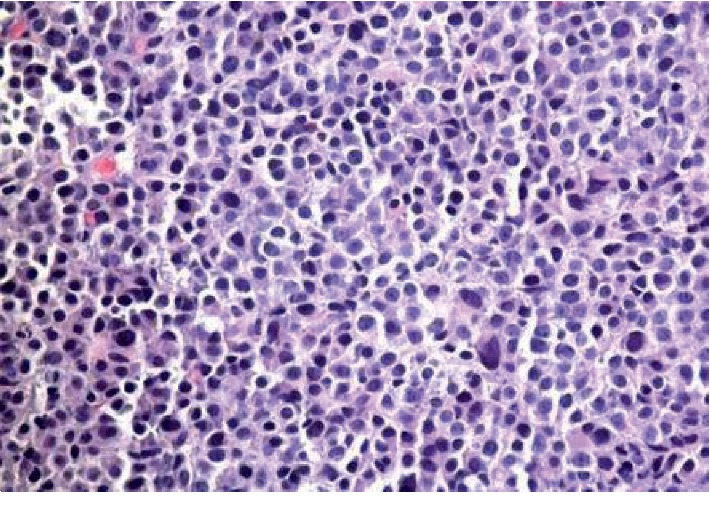
H&E (haematoxylin and eosin) stain of the biopsied soft tissue sternal mass shows soft tissue is diffusely infiltrated by large sheets of large cells with plasmacytoid morphology.

**Figure 4:**
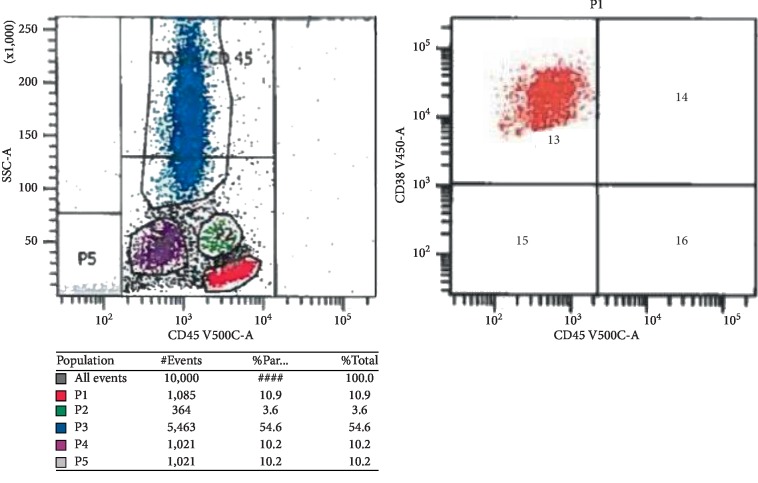
Flow cytometric analysis reveals a plasma cell population (P4) with higher side scatter (SSC). The plasma cells are positive for CD38^bright^, cytoplasmic lambda, and CD56 and negative for CD 19.

Flow cytometry on the biopsied specimen revealed an immunophenotype of monotypic plasma cells (11% of cells) positive for cytoplasmic lambda, CD38, CD56, dim CD45, and CD117 and negative for CD19 and CD20 with morphology showing atypical plasma cells with high nucleus-to-cytoplasm ratio, eccentric and irregular nucleuses, and frequent mitosis (Figures 3 and 4). Cytopathology immunohistochemical (CD20, CD117, cyclinD1, c-MYC, and p53) and in situ hybridization (EBR) stains showed neoplastic cells positive for c-MYC, CD56 and CD117 and negative for CD19, CD 20, cyclinD1, p53, and EBER. FISH using probe and location MYC (8q24) was negative for MYC rearrangement. A full multiple myeloma workup was then pursued. Serum free light chain analysis showed kappa light chain of 0.04 mg/dL, lambda light chain of 213 mg/dL, high IgG levels of 1840 mg/dL with an IgG lambda monoclonal band on urine and serum immunofixation, and low beta-2 microglobulin 0.5 mg/L.

According to the 2016 International Myeloma Working Group criteria, this patient met criteria for multiple myeloma with a biopsy proving soft tissue extramedullary plasmacytoma and multiple myeloma defining events with osteolytic lesions shown on imaging and a free light lambda chain ratio >100 mg/L and elevated LDH. The patient was diagnosed with stage III plasma cell IgG lambda multiple myeloma [12, 13]. The patient clinically had acute disease progression and severely worsening neurological symptoms; a bone marrow biopsy was deferred. A lumbar puncture was done and showed no malignant plasma cells in the CSF. She received palliative radiation therapy to the skull base, thoracic spine, and sacrum, and systemic treatment was started.

Cyclophosphamide 600 mg/m^2^, bortezomib 1.3 mg/m^2^, and dexamethasone 40 mg (CyBorD) regimen was initially given [14, 15]. An attenuated dose of cyclophosphamide was used with an attempt to quickly debulk the disease. Unfortunately, the disease continued to progress, the patient had worsening jaw pain, within 18 days after starting CyBorD. Repeat CT of the maxillofacial area revealed enlargement of the left masticator space lesion. Her treatment regimen was changed to second-line therapy with dexamethasone 40 mg, cyclophosphamide 400 mg/m^2^, etoposide 40 mg/m^2^, and cisplatin 10 mg/m^2^(DCEP) [16]. Seven days later, with symptoms of dysphagia and lower extremity weakness, the decision was made to add daratumumab 16 mg/kg for primary refractory myeloma [17, 18].

After addition of daratumumab, she began to clinically improve. Subsequent CT of the maxillofacial showed a decrease in previously seen masticator space masses (Figure 1(b)). Furthermore, there were decreased size paraspinal and epidural lesions on CT lumbar and thoracic spine (Figure 2(b)). The Chest, abdomen, and pelvis CT scan also demonstrated substantial interval decreased size of right chest wall and left hemisacrum soft tissue masses. Based on the International Myeloma Workshop Group criteria [19], the patient had a partial response to one dose of daratumumab, with CT imaging showing ≥50% reduction in size of soft tissue plasmacytoma and marked reduction in lambda light chain levels from 213 to 3.02 mg/dL. The main toxicity during the hospital course was neutropenia with fever and a nadir ANC 500. Moreover, repeat MRI brain and spine 17 days later significantly decrease in enhancing soft tissue in the lumbar extradural space, with the resolution of compression upon the cauda equina at the levels of L3-L4 and L4-L5 as described above. Persistent soft tissue in the left L3-L4, L4-L5, and L5-S1 neural foramina, encasing/compressing the left L3, L4, and L5 nerve roots was found. Slight improvement of large bulky bilateral sacral masses was observed, which extends to the left presacral and left sciatic foramen regions. Persistent obliteration of the left sacral foramina was observed. Stable mild superior endplate pathologic compression deformities at T12, L4 and L5 were also observed. In the brain MRI, the bony lesions overall appears smaller in size and less bulky, most consistent with a favorable response to treatment. No evidence for brain parenchymal metastatic disease, acute infarct, or acute hemorrhage. A repeat flow cytometry was done 30 days into her admission of her left iliac, but cytopathology analysis was done and showed from the core biopsy with paucicellular and a few plasma cells and rare plasmacytoid cells of uncertain lineage (erythroid versus plasma cellular), amidst scattered bone marrow elements, fibrous tissue, and abundant blood. Immunostains were performed and show rare CD138 staining plasma cells, abundant CD3 staining T cells. Kappa and lambda are noncontributory due to depletion of the tissue upon deeper sectioning. Cam 5.2 is negative and excludes any epithelial cells. She continued to improve, and her ANC rose above 1000 over the next few days. She began moving her lower extremities and was able to walk with crutches at the time of discharge.

## 3. Discussion

This is a rare case of a very young patient presenting with neurological symptoms found to have multiple myeloma associated with multiple soft tissue extramedullary plasmacytomas. This case has multiple atypical aspects including the patient's age, presenting symptoms, and number and locations of extramedullary plasmacytomas. The median age of diagnosis of multiple myeloma is 69 years with only 15% of cases occurring in those less than 55 years [1]. Multiple myeloma occurs slightly more in men with typical symptoms of fatigue, anemia, bone pains, and/or hypercalcemia with renal insufficiency (CRAB features). The neurological complications, usually radiculopathy and peripheral neuropathies, are associated with spinal cord compression or neural compression secondary to bone damage.

The patient in this case however presented with atypical neurologic findings often seen in CNS disease. She had diplopia and ataxia along with paraplegia. The location of her plasmacytomas resulted in these unusual neurologic findings. In addition, given her high disease burden, her labs were not consistent with the typical CRAB features, and in fact the beta-2 microglobulin was low. Her IgG serum level was mildly elevated. The bulk of her measurable disease in the serum was noticed by the elevated serum lambda light chain of 213 mg/dL. Given the extent of bone damage, she had an oligosecretory myeloma, which is rare.

Another unique presentation of this case is the large number of soft tissue extramedullary plasmacytomas. Extramedullary plasmacytomas have an incidence of 0.04 cases per 100,000 individuals and usually are solitary masses with 80–90% arising from mucosal associated lymphoid tissue in the upper airways including nasopharynx and paranasal sinuses [20]. Furthermore, only 1-2% of patients at the time of initial diagnosis have extramedullary disease, and 8% develop extramedullary disease later on [21]. They also commonly present with obstruction of the nasal passages, masses in the palatal or maxillary, and with erosions leading to epistaxis and facial pain [20–22]. In this patient, she had multiple soft tissue masses throughout her chest wall, left pelvis, and thoracic spine along with a skull-base mass with intracranial and intraorbital extension. There have been only a few case reports of intracranial involvement with either multiple myeloma or intracranial plasmacytomas, but this level of intracranial involvement and associated chest and abdominopelvic involvement in one patient has not been reported in the literature [21–25]. The clinical scenario is suggestive of a possible aggressive form of oligosecretory myeloma which mainly progresses as plasmacytomas throughout the body, with minimal measurable disease present in the serum.

Despite progress in effective treatment, multiple myeloma remains incurable. The patient not only failed to respond to initial treatment with CyBorD regimen but also she progressed acutely while on therapy. Second-line therapy with DCEP regimen was ineffective acutely until daratumumab was added on and then she had a notable response.

The efficacy of daratumumab in treatment of multiple myeloma has been well documented over the past decade. Daratumumab is a monoclonal antibody to CD38 and widely expressed by several hematopoietic cells in the bone marrow, most notably plasma cells. Of note, it is also expressed on osteoclasts and bronchial epithelial cells [26]. It has been shown in preclinical studies that daratumumab kills myeloma cells by both antibody-dependent cellular cytotoxicity and phagocytosis by blocking the inhibitory signals from CD38, thus boosting cytotoxic T cells [27–29]. In its first clinical trial, SIRIUS, daratumumab was used in relapsed refractory myeloma in a multicentered phase 2 trial. A majority of these patients (80%) had received autologous stem-cell transplants and were refractory to proteasome inhibitors and immunomodulatory drugs (95%) and received a median of five previous lines of therapy. The 12-month overall survival was 64.8%, and median overall survival with 17.5 months was 29.2% showing overall response with 30% of the patients responding to daratumumab [18]. In 2015, it was initially approved by the FDA for treatment of patients with refractory multiple myeloma who had failed 3 or more prior lines of therapy [30]. In the CASTOR and POLLUX trial, both phase 3, randomized, multicenter, open label trials involving adding daratumumab for relapsed or refractory patients (who had already received one or more previous lines of therapy (autologous stem-cell transplant (ASCT), alkylating agents and proteasome inhibitors). Both trials showed significantly increased response rates and progression-free survival by more than 60% with the addition of daratumumab [31, 32].

Since then, daratumumab has been approved, in combination with proteasome inhibitors (PIs) and immunomodulatory drugs (IMiDs) in the upfront setting for newly diagnosed myeloma patients. These clinical trials have shown daratumumab to work synergistically with IMiDs and PIs with high potency and efficacy in killing myeloma cells with tolerable toxicity profiles [26, 31–36].

The ALCYONE trial was the first phase 3 daratumumab clinical trial to gain FDA approval for newly diagnosed multiple myeloma patients who were ineligible for autologous stem-cell transplantation. The patients were randomized to either nine cycles of bortezomib, melphalan, and prednisone alone (control arm) or with daratumumab (experimental arm) until disease progression. Patients in the daratumumab group had significantly longer progression-free survival at 18 months at 71.6% vs. 50.2% in the control group. The overall response in the daratumumab group was 91% with 22.3% negative for minimal residual disease (MRD) compared to 74% overall response and 6.2% negative for MRD in the control group [33]. MRD was defined by 1 tumor cell per 10^5^ white cells.

Finally, the MAIA trial was done by comparing either daratumumab and lenalidomide with dexamethasone or lenalidomide with dexamethasone in multiple myeloma patients who were ineligible for autologous stem-cell transplant. Those in the daratumumab group had disease progression/death in 26.4% compared to 38.8% with lenalidomide and dexamethasone arm. Patients with daratumumab had 30 months without disease progression and were alive 70.6% in the experimental arm compared to 55.6% to the control arm [34]. A complete response or better was achieved in 47.6% of patients in the daratumumab group compared to 24.9% in the control group. In terms of MRD, 24.2% of patients in the daratumumab arm compared to 7.3% in the control arm had evidence of minimal residual disease [34]. Throughout all these trials the most common manageable toxicities of daratumumab were neutropenia, thrombocytopenia, increased respiratory infections, and infusion-related reactions.

Based on these clinical trials, daratumumab is highly potent and effective in the treatment of myeloma patients in combination with PIs and IMiDs, but for the advanced myeloma patient with high disease burden such as our patient, often the initial therapy for rapid disease control remains combination therapies such as CyBorD, DCEP, or other such heavy chemotherapy-based regimen. In this case, the availability of PIs and IMIDs was limited due to the inpatient setting. Additionally, at the time of the actual events, daratumumab was only FDA approved in the relapsed refractory multiple myeloma. Therefore, the choice for use of CyBorD induction with escalation to DCEP and daratumumab added once deemed refractory. This case suggests that not only would daratumumab be option for upfront therapy with PIs/IMiD combinations but also with multidrug chemotherapy regimens such as DCEP.

This case report shows a unique regimen of DCEP plus daratumumab resulting in a robust initial response, after the failure of CyBorD. This case report suggests that daratumumab in combination with DCEP may be another option to use for inpatient treatment of primary refractory and aggressive multiple myeloma disease. Especially, given the real world delays in obtaining IMiD/PI therapy for acutely ill hospitalized patients, this case also highlights how very different the presentation of multiple myeloma may be in a very young patient compared with older patients.

## 4. Conclusion

This provides insight into not only how young patients may present aggressively but also a potential way to manage it. The risk of multiple myeloma increases with age, but this is a case report of a patient who was diagnosed with a large amount of disease burden at a young age showing that young patients may present more aggressively. This case highlights that multiple myeloma should be included in the differential diagnosis of a young patient with neurological symptoms and mass lesions, after more common causes have been ruled out. Moreover, this patient has an aggressive form of multiple myeloma presenting with several plasmacytomas and neurological symptoms. She progressed and failed induction with CyBorD, and only after switching to DCEP with addition of daratumumab, did she have a partial response. The response was long enough to stabilize and discharge her from the hospital safely.

Further research is needed in this field to determine optimal treatment regimens for this subset of young patients. These cases are rare, thus hampering the use of clinical trials in these patients. Therefore, case reports and registries may be needed to help collect data for future analysis and studies. Ideally these patients would be most likely to benefit from a cure, given the young age and potential loss of life years.

## Additional Points

*Clinical Significance*. It is important to recognize that multiple myeloma can occur in very young patients and may present and respond differently in this younger-aged cohort. Diagnosing and treating this group can be challenging, and it is crucial to identify optimal initial treatment regimens for these patients. A great way to gather this information would be by creating a registry.
